# An adaptive fuzzy prediction model for real time tumor tracking in radiotherapy via external surrogates

**DOI:** 10.1120/jacmp.v14i1.4008

**Published:** 2013-01-07

**Authors:** Ahmad Esmaili Torshabi, Marco Riboldi, Abbas Ali Imani Fooladi, Seyed Mehdi Modarres Mosalla, Guido Baroni

**Affiliations:** ^1^ Department of Electrical and Computer Engineering Kerman Graduate University of Technology Kerman Iran; ^2^ TBMLab‐Department of Bioengineering Politecnico di Milano Milano Italy; ^3^ Applied Microbiology Research Center Baqiyatallah University of Medical Sciences Tehran Iran

**Keywords:** radiotherapy, tumor tracking, fuzzy logic, data clustering, adaptive prediction model

## Abstract

In the radiation treatment of moving targets with external surrogates, information on tumor position in real time can be extracted by using accurate correlation models. A fuzzy environment is proposed here to correlate input surrogate data with tumor motion estimates in real time. In this study, two different data clustering approaches were analyzed due to their substantial effects on the fuzzy modeler performance. Moreover, a comparative investigation was performed on two fuzzy‐based and one neuro‐fuzzy–based inference systems with respect to state‐of‐the‐art models. Finally, due to the intrinsic interpatient variability in fuzzy models' performance, a model selectivity algorithm was proposed to select an adaptive fuzzy modeler on a case‐by‐case basis. The performance of multiple and adaptive fuzzy logic models were retrospectively tested in 20 patients treated with CyberKnife real‐time tumor tracking. Final results show that activating adequate model selection of our fuzzy‐based modeler can significantly reduce tumor tracking errors.

PACS number: 87

## I. INTRODUCTION

In external beam radiotherapy the final purpose is to produce 3D dose coverage onto the tumor volume while minimizing the dose to the surrounding healthy tissues. When tumor motion is an issue, the delivered dose does not match with the planned treatment, resulting in some over‐ and underdosage in the tumor volume, as a function of motion magnitude and frequency.^(^ ^1^ ^)^ Various strategies have been developed which can be applied to compensate the effects of intrafractional motion, including breath holding, respiratory gating, and tumor tracking.^(^ ^2^ ^–^ ^9^ ^)^ In this latter case, the exact information of tumor position in real time is required during treatment.^(^ ^10^ ^)^ The accuracy of real time tumor motion localization strongly depends on breathing motion variability during consecutive breathing cycles.^(^ ^11^ ^–^ ^13^ ^)^ There are several strategies to obtain tumor position information over time, ranging from fluoroscopic imaging^(^ ^7^ ^–^ ^8^ ^)^ to the use of external surrogates.^(^ ^12^ ^–^ ^15^ ^)^ In fluoroscopy‐based approaches, although the exact tumor location can be captured at each frame, some concerns arise regarding the additional imaging dose received by patients.^(^ ^16^ ^)^ In radiotherapy with external respiratory surrogates, tumor motion is tracked using a consistent correlation between external surrogates and internal implanted clips, as detected by stereoscopic X‐ray imaging. In this way, tumor position is estimated by means of a correlation model, even though there are uncertainties in generating a consistent correspondence between external/internal data to infer tumor motion as a function of time.

Recently, several external/internal correlation models have been developed.^(^ ^11^ ^,^ ^17^ ^–^ ^22^ ^)^ Some of these models were taken into account as a comparative study in a recent work.^(^ ^23^ ^)^ In these models, tumor motion (model output) is correlated with rib cage/abdomen motion, provided as model input. In this study, we further investigate correlation models based on fuzzy logic.^(^ ^24^ ^–^ ^28^ ^)^ Fuzzy logic‐based correlation was selected due to the reduced dependency to precise input information versus conventional mathematical models. Recently, the improved performance of fuzzy logic‐based systems in data analysis causes arising applications of fuzzy logic theory in many fields.^(^ ^29^ ^–^ ^31^ ^)^ Since the uncertainty in patient breathing results in a huge variability in terms of input/output dataset, a fuzzy environment may be optimal to correlate input data with tumor motion estimation.^(^ ^17^ ^)^

In the construction of the fuzzy inference systems, data clustering plays an important role.^(^ ^32^ ^–^ ^33^ ^)^ Data clustering partitions the dataset into several groups in order to create homogeneous clusters for better data analysis. Our fuzzy model consists of two different data clustering algorithms: 1) subtractive‐based, and 2) fuzzy C‐means (FCM)‐based.^(^ ^34^ ^–^ ^37^ ^)^ In the subtractive clustering algorithm, each data point of the dataset can potentially be candidate as cluster center proportionally to the density of surrounding data points, whereas the FCM algorithm uses fuzzy grouping properties such that the given data point in a dataset belongs to several clusters with different membership degrees. Fuzzy logic models can also be combined with neural networks in a mixed adaptive neuro‐fuzzy inference system (ANFIS).^(^ ^38^ ^)^

Three different fuzzy‐based external/internal correlation models based on (i) subtractive clustering, (ii) FCM clustering, and (iii) ANFIS systems were implemented and comparatively tested. The generated models can be updated automatically, using new arrived training data points during treatment. Each correlation model is able to track tumor motion independently, and different motion estimations are calculated according to each modeler characteristics and also to the degree of variability of external/internal motion data.

Since each patient has a unique breathing pattern and may also be affected by a different degree of variability, a model selectivity option is proposed here in order to select the best correlation model in the training step on a case‐by‐case basis.

The implemented strategies were compared together and also with a typical CyberKnife Synchrony module in terms of residuals targeting errors in 20 patients.^(^ ^12^ ^,^ ^14^ ^–^ ^15^ ^)^

## II. MATERIALS AND METHODS

### A. CyberKnife system and patient database

Among the external radiotherapy devices, the Synchrony respiratory tracking system integrated with the CyberKnife robotic linear accelerator (Accuray Incorporated, Sunnyvale, CA) is an implementation of real time tumor tracking. In this system, tumor motion is tracked by using a correlation model generated between the synchronized signals coming from external surface markers and internal implanted clips near or inside the tumor region.^(^ ^14^ ^)^ The external signals are obtained by monitoring three external markers placed on a vest, using an infrared tracking system (at ~25 Hz). Internal signals depicting tumor location are extracted through orthogonal X‐ray imaging, where implanted clips are automatically segmented. The correlation model is built at the beginning of each irradiation session and updated as needed over the course of treatment with intermittent X‐ray imaging. The accuracy of Cyberknife Synchrony measured in real patient treatments is reported (1 SD) within 1.9 mm (SI), 1.9 mm (LR), and 2.5 mm (AP) in terms of pure external/internal correlation errors.^(^ ^15^ ^)^ The Synchrony module models instantaneous tumor motion as a function of the external marker coordinates, and predicts tumor motion in the near future to account for the system lag (~115−192.5 ms).^(^ ^12^ ^,^ ^15^ ^)^ Retrospective clinical studies show that the uncertainties related to prediction in the future are of smaller magnitude than the ones due to external/internal correlation modeling.^(^ ^15^ ^)^ As the treatment proceeds, relevant data including tumor motion model and prediction, the coordinates of external markers, and intermittent clip localization, are logged and stored in ASCII format.^(^ ^15^ ^)^

The performance of Synchrony respiratory tracking was analyzed in terms of residual tracking errors measured with X‐ray imaging during treatment. The same group of patients that was selected in our previous work^(^ ^23^ ^)^ was used for the analysis: 10 worst cases and 10 control cases, randomly selected among the population. Case selection was carried out in order to include both critical cases in terms of residual correlation error (worst group) and examples that are easily handled by current clinical solutions (control group).^(^ ^23^ ^)^

### B. Development of fuzzy correlation model

The concept of Fuzzy Logic was proposed in 1965 by Lotfi A. Zadeh.^(^ ^24^ ^–^ ^28^ ^)^ Basically in fuzzy logic, linguistic variables are used to represent operating parameters in order to apply a more human‐like way of thinking. Fuzzy logic incorporates a simple IF–THEN rule‐based approach to solve a problem, rather than attempting to model a system mathematically, and this property plays a central role in most of fuzzy logic applications. More recently, fuzzy logic has been highly recommended to generate solutions for problems based on qualitative, incomplete or imprecise information, where rigorous, analytical solutions do not exist. The main idea of fuzzy systems is to extend the classical two‐valued modeling of concepts and attributes in a sense of gradual truth. It is currently used in the fields of business, systems control, electronics, traffic engineering, and weather forecasting, to yield superior results compared to conventional algorithms.

**Figure 1:**
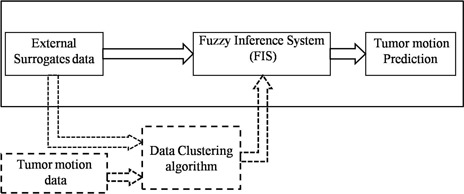
Block diagram of fuzzy inference system (upper part) and data clustering algorithm (lower part).

Alternatively, fuzzy logic‐based systems require membership functions as a representation of the magnitude of participation for each input. The proposed fuzzy correlation model involves data clustering^(^ ^33^ ^–^ ^34^ ^)^ for membership function generation, as a requirement for the fuzzy inference system section (Fig. 1, lower dashed rectangle). After system configuration, when a data point is given as input, the fuzzy inference system operates by: 1) data fuzzification, 2) if–then rules induction, 3) application of implication method, 4) output aggregation, and 5) defuzzification steps. (Fig. 1, upper solid rectangles).^(^ ^26^ ^–^ ^28^ ^)^

In the implemented fuzzy logic algorithm, data from all three external markers were used in an input matrix with nine columns as input, where columns represent the x(t), y(t), and z(t) of each marker.

**Figure 2:**
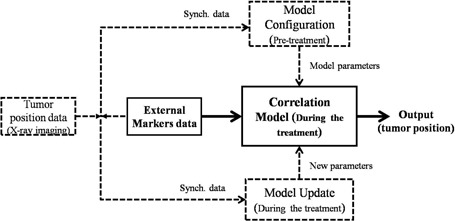
Flowchart of correlation model depicting model configuring (upper part), model performance (middle part), and model updating (lower part).

The fuzzy correlation model was implemented in MATLAB (The MathWorks Inc., Natick, MA) using the fuzzy logic toolbox. The model is built at the beginning of each irradiation session using the same training data considered by the Synchrony module (Fig. 2, upper part). Training data include 3D external markers motion as model input and internal implanted markers as model output in a synchronized fashion. The number and time of training data collection range from 4 to 27 and 58 to 603 sec, respectively, for all patients. After model parameters are estimated, the model is applied to infer tumor trajectory as a function of time during treatment, relying on external markers data only (Fig. 2, middle part). The model can be updated as needed over the course of treatment with intermittent X‐ray imaging representing the internal marker location, synchronized to external markers data (Fig. 2, lower part).

As seen in Fig. 1, in the construction of the fuzzy inference systems data clustering plays an important role.^(^ ^32^ ^–^ ^33^ ^)^ Data clustering analysis is the organization of a collection of datasets into clusters based on similarity. Two of the most representative techniques of data clustering were utilized in our model: subtractive clustering and fuzzy C‐means clustering.

In the training step, two fuzzy inference systems based on the above clustering approaches and also on an adaptive neuro‐fuzzy inference system are configured for motion prediction during treatment. The properties and the implementation of these inference systems are detailed in the following paragraphs.

### B.1 Fuzzy inference system using subtractive clustering

In this model, the subtractive clustering algorithm^(^ ^36^ ^)^ was considered in initial data grouping and membership function generation. In subtractive clustering, each data point of the dataset can potentially be candidate as cluster center, proportionally to the density of neighboring data points. Therefore, based on the following equation, a calculation is performed to define the cluster center:

(1) $$D_{j}=\sum_{i=1}^{N}\text{exp}\left(-\frac{{\left\|x_{i}-c_{j}\right\|}^{2}}{{\left(r/2\right)}^{2}}\right)$$

where $x_{i}$ is the ith measured data point, $c_{j}$ is the center of the cluster, and *r* is the neighborhood radius or influence range.

In this algorithm, a small influence range yields many small clusters in the dataset and specifying large influence range results in few large clusters. In the implemented model, the value of such influence range was set to one‐third of the width of the training data space, according to the same approach followed in our previous work.^(^ ^23^ ^)^

By the end of data clustering, a set of fuzzy rules and membership functions are extracted. Each cluster represents a rule. Since the membership functions used for our fuzzy inference system are in Gaussian shape, the cluster centers and the distance between them can be used as required parameter for membership function generation over the input and output training dataset.

### B.2 Fuzzy inference system using FCM clustering

The fuzzy C‐means algorithm uses fuzzy partitioning for data clustering, such that each data point of the dataset belongs to several groups with specific membership grades between 0 and 1. The membership grade represents the relationship of a data point at each cluster, and its magnitude depends on the distance of given data point to the cluster centers. It should be noted that in this way, before employing the FCM technique, the training dataset is clustered into *n* groups using the subtractive clustering algorithm, as mentioned above.

From the mathematical point of view, membership functions in FCM clustering are obtained by minimization of the following metric:

(2) $$J_{m}=\sum_{i=1}^{N}\sum_{j=1}^{C}u_{ij}^{m}{\left|x_{i}-c_{j}\right|}^{2}$$

where *m* is any real number greater than 1, $u_{ij}$ is the grade of membership of *x* in cluster *j*, $x_{i}$ is the ith measured data point, and *c* is the center of the cluster. At first, FCM assumes the mean location of each cluster as initial cluster center. Next, the FCM algorithm assigns every data point a membership degree for each cluster, and then iteratively moves the cluster centers *c* and updates the membership degrees $u_{ij}$:

(3) $$J_{m}=\sum_{i=1}^{N}\sum_{j=1}^{C}u_{ij}^{m}{\left|x_{i}-c_{j}\right|}^{2}$$

(4) $$c_{j}=\frac{\sum_{i=1}^{N}u_{ij}^{m}\cdot x_{i}}{\sum_{i=1}^{N}u_{ij}^{m}}$$

This iteration will stop when $\left|U^{\left(k+1\right)}-U^{\left(k\right)}\right|<\varepsilon$, where ε is a termination criterion between 0 and 1, *U* is the matrix of [$u_{\text{ij}}$] and *k* is the number of iterations.

The structure of the two above fuzzy inference systems used for our predication model is based on the Sugeno (or Takagi‐Sugeno‐Kang) model.^(^ ^39^ ^)^ Since this model is computationally more efficient and gives a faster response, its implementation is proper here for our real‐time motion estimation.

### B.3 Adaptive neuro‐fuzzy inference system(ANFIS)

ANFIS is an adaptive network which uses neural network learning algorithms and fuzzy inference system to associate the external marker motions as input to the tumor motion as output. In ANFIS, the membership function parameters are determined using subtractive clustering in the fuzzy section, and then adjusted using either a back‐propagation algorithm alone or in combination with a least squares type method.

As a brief description about the ANFIS structure, if x and y are assumed as input variables for single‐output fuzzy inference system, the output variable is obtained by applying fuzzy rules to a fuzzy set of input variables. The rule set contains two fuzzy if‐then rules for the first order Sugeno fuzzy model as follows:

(5) $$\text{Rule}\;1:\;\text{if}\;x\;\text{is}\;A1\;\text{and}\;y\;\text{is}\;B1\;\text{then},\;f_{i}=p_{1}x+q_{1}y+r_{1}$$

(6) $$\text{Rule}\;2:\;\text{if}\;x\;\text{is}\;A2\;\text{and}\;y\;\text{is}\;B2\;\text{then},\;f_{2}=p_{2}x+q_{2}y+r_{2}$$

where $p_{i}$, $q_{i}$, and $r_{i}$ (i=1 or 2) are linear parameters, and $A_{i}$, $B_{i}$ are linguistic labels characterized by appropriate membership functions.^(^ ^38^ ^)^ The Gaussian and Bell‐shape membership functions are increasingly popular for specifying fuzzy sets due to the smoothness and concise notation. The architecture of ANFIS consists of five layers, and the number of neurons at each layer equals to the number of rules. Layer 1 consists of input nodes. Each node generates membership grades using membership functions. In Layer 2 which includes rule nodes, the AND operator is used in order to product all incoming signals that represents the firing strength for that rule ($w_{i}$). In the third layer, the purpose is to calculate the ratio of each ith rule's firing strength to the sum of all rules' firing strength. Thus the output is called normalized firing strength ($w_{i}$). The fourth layer computes the contribution of each rule toward the total output. In layer 5, the single output node calculates the overall output by summing all incoming signals:

(7) $$f=\frac{w_{1}f_{1}+w_{2}f_{2}}{w_{1}+w_{2}}=\bar{w_{1}}f_{1}+\bar{w_{2}}f_{2}$$

In ANFIS, the nonlinear parameters ($a_{i}$, $b_{i}$, and $c_{i}$) associated with the membership functions are tuned through the learning process in an iterative fashion. The parameter tuning process is controlled proportionally to the error value of the fuzzy inference system section in correlating the input/output dataset.

All three correlation models can be updated automatically, using X‐ray imaging points during the treatment. The updating step was performed by adding the last imaging data to the previous data and then rebuilding the correlation models using all the gathered imaging data.

### C. Model selectivity algorithm

The produced correlation models can predict the tumor motion independently. Although all three modelers track tumor motion in a same trajectory, they differ in their prediction accuracy due to their specifications in membership functions generation and also input/output variability. In other words, the performance of each modeler is not necessarily most accurate for all patients. Thus selecting an adaptive fuzzy model may be useful for each patient uniquely. Consequently a model selectivity option was implemented in order to select automatically the best performing correlation model in the training step for each patient.

**Figure 3:**
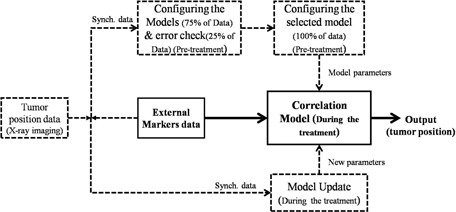
Model selectivity option in training step of the final flowchart.

For this purpose, the available training dataset was divided into two parts with one‐fourth ratio. Three‐quarters of the training dataset was used as training data for three modelers configuration, whereas the rest of the dataset (25%) was exploited to check the accuracy of each modeler. The optimal modeler for each patient was selected relying on the preliminary check performed on 25% of the training dataset, assuming that optimal performance during training resulted as optimal during the rest of the treatment. Figure 3 represents the flowchart of our final prediction model, including the model selectivity subroutine during training (configuring the models and error check, pretreatment). It should be considered that a large amount of training dataset contains all the necessary representative features and, therefore, the process of selecting the proper modeler is easier. The investigation of model selectivity option was performed using our patient database.

## III. RESULTS

**Figure 4:**
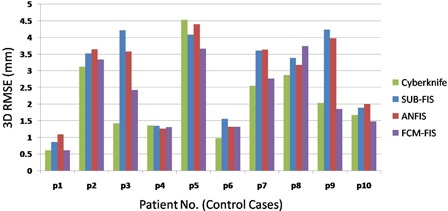
RMSE vs. patient numbers (control group).

**Figure 5:**
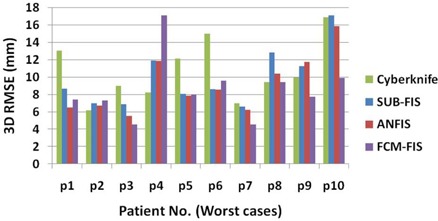
RMSE vs. patient numbers (worst group).

Figures 4 and 5 represent the RMSE (route mean square error) of 3D targeting errors of different tumor tracking models for control and worst patient groups, respectively. It should be noted that in these figures, SUB−FIS=SUBtractive−based Fuzzy Inference System, and FCM−FIS=Fuzzy C‐Means‐based Fuzzy Inference System.

The statistical analysis (Kolmogorov‐Smirnov test) revealed that 3D targeting errors are not normally distributed at 99% confidence: hence, nonparametric statistical tests were used. The median and interquartile range of total 3D targeting errors overall 10 patients in control and worst groups are shown in Table 1, in order to have a comparative investigation between correlation models performance.

**Table 1:** The median and interquartile range of 3D RMSEs overall 10 patients in different modelers in control and worst groups.

	*Control Group*		*Worst Group*	
*Modeler*	*Median*	*Interquartile Range*	*Median*	*Interquartile Range*
CyberKnife	1.850	1.421	9.669	4.401
SUB‐FIS	3.457	2.322	8.624	4.499
ANFIS	3.377	2.151	8.174	4.868
FCM‐FIS	2.137	1.836	7.820	2.243

Paired wise statistical comparison (Friedman test) showed no significant difference among the different algorithms for the control group, whereas substantial variations were confirmed for the worst group (p‐value <1%). A posthoc analysis based on ranks highlighted a significantly worse performance for SUB‐FIS and ANFIS if compared to the other strategies; conversely, the mean ranks for FCM‐FIS and CyberKnife can be considered comparable.

Results show a significant interpatient variability in both groups, as confirmed by the Kruskal‐Wallis test (p‐value <1%). As depicted for the worst group in Fig. 5, for patient 4 the FCM‐FIS performance is the worst, while the SUB‐FIS is the best, whereas for patient 10 the FCM performance is the best with a huge error reduction improvement, while the SUB‐FIS is the worst. Thus, the model selectivity test is put forward for selecting the proper modeler for each patient using the training dataset. Table 2 shows the selected modeler for each case in the control and worst groups.

**Table 2:** Selected modeler vs. the best modeler in training step for patients in the control and worst groups. The (√) and (x) symbols represent the correct and incorrect selections, respectively.

	*Control Group*		*Worst Group*	
	*Selected Model*	*Best Model*	*Selected Model*	*Best Model*
P1	SUB‐FIS	FCM‐FIS (x)	ANFIS	ANFIS (√)
P2	FCM‐FIS	FCM‐FIS (√)	ANFIS	ANFIS (√)
P3	SUB‐FIS	FCM‐FIS (x)	FCM‐FIS	FCM‐FIS (√)
P4	FCM‐FIS	ANFIS (x)	ANFIS	ANFIS (√)
P5	SUB‐FIS	FCM‐FIS (x)	ANFIS	ANFIS (√)
P6	FCM‐FIS	FCM‐FIS (√)	FCM‐FIS	ANFIS (x)
P7	ANFIS	FCM‐FIS (x)	ANFIS	FCM‐FIS (x)
P8	ANFIS	ANFIS (√)	FCM‐FIS	FCM‐FIS (√)
P9	FCM‐FIS	FCM‐FIS (√)	FCM‐FIS	FCM‐FIS (√)
P10	FCM‐FIS	FCM‐FIS (√)	FCM‐FIS	FCM‐FIS (√)

The median and interquartile range of 3D errors overall 10 patients using model selectivity test vs. typical CyberKnife modeler are reported in Table 3 for control and worst groups, respectively.

**Table 3:** The median and interquartile range of 3D RMSEs overall 10 patients when the model selectivity test is active.

	*Control Group*		*Worst Group*	
*Modeler*	*Median*	*Interquartile Range*	*Median*	*Interquartile Range*
CyberKnife	1.850	1.421	9.669	4.401
Fuzzy model using selectivity option	2.513	2.280	7.761	3.010

As seen in Table 3, activating the model selectivity option in control group results in a worst performance in comparison with FCM‐FIS (Table 1), but in the worst group an improvement in error reduction can be achieved. It is obvious also by comparing the selected modeler in the training step depicted in Table 2 with the final modeler performance shown in Fig. 5 patient by patient. The selected modeler in the training step was typically not correct for patients belonging to the control group. But the decisions for worst group are 80% correct, where the most concerns arise due to the high RMSE values. During the modeler selection process, the selectivity option is typically correct when large differences among different models can be measured. While the performances of all three modelers are not highly dissimilar, the selected modeler may not be the optimal one, but the prediction of tumor motion is in an acceptable range.

**Figure 6:**
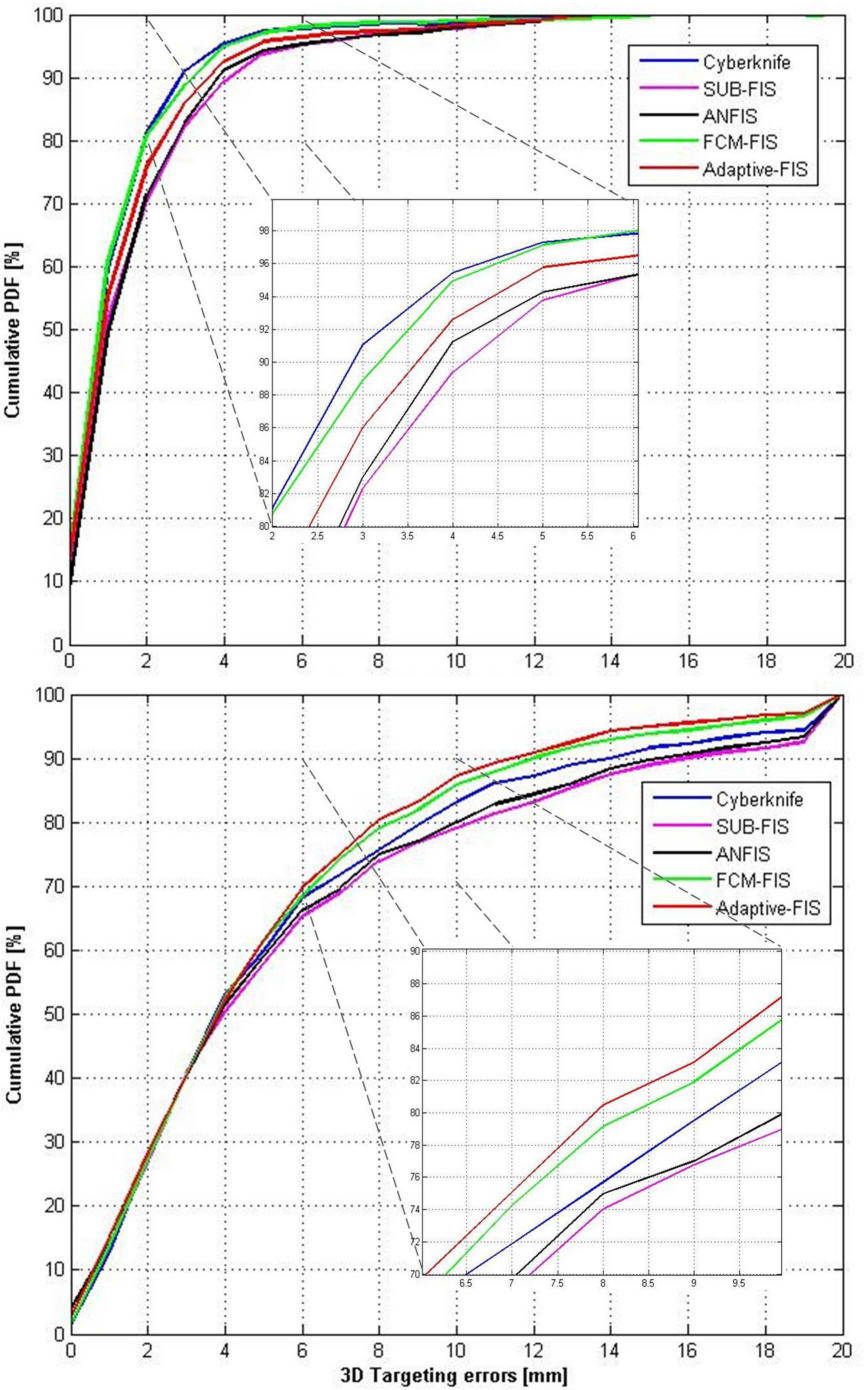
Cumulative probability distribution functions (PDFs) of 3D targeting errors for worst (bottom) and control (top) groups.

Figure 6 represents cumulative probability distribution functions (PDFs) of the 3D targeting errors for worst and control groups.

Each PDF takes into account all the imaging points acquired during treatment for model validation and update. As visible in this figure, the best performance for the worst group is achieved by using the adaptive fuzzy modeler when model selectivity option is active. This ensures better performance, especially for larger errors (i.e., targeting residuals beyond 6 mm (Fig. 6).

## IV. DISCUSSION

The input/output database was extracted from a large patient database and allowed the quantitative comparison between proposed strategies and CyberKnife Synchrony. These latter were selected in order to include both critical (worst patients) and average (control patients) working conditions for external/internal correlation algorithms. As inputs are received by the model, the number of clustering is determined; then, the required membership functions and rules are extracted, which have strong effects on the proved performance of the model.

Three fuzzy correlation models were investigated to correlate external breathing motion as input data with tumor motion estimation: subtractive‐based fuzzy correlation model, FCM‐based fuzzy correlation model, and adaptive neuro‐fuzzy inference system

The two former correlation models were selected in order to investigate the effect of data clustering techniques on membership function generation, that have an effective role on final modeler performance. On the other hand, a comparative investigation was performed considering adaptive neuro‐fuzzy inference system with two former modelers. Finally, a model selectivity option was proposed to select an adaptive modeler for each patient uniquely, relying on training data to identify the best performing strategy. This is meant to take into account that each patient has a unique pattern in breathing and also a different degree of uncontrolled motion (mainly patients with respiratory disorder). Through this algorithm, the best model may be found for each patient on a case‐by‐case basis, relying on a fully automated strategy for the selection of the optimal correlation.

Results show that the performance of different algorithms on the control group is not statistically different, thus indicating that the implemented fuzzy models are able to reach an adequate accuracy. Conversely, significant differences were found among the worst cases group, with SUB‐FIS and ANFIS exhibiting degraded performance if compared to other strategies. The cumulative PDF in this case shows that the FCM‐FIS algorithm may ensure a benefit in terms of larger errors reduction (Fig. 6).

Among the fuzzy models, the implemented approximately 90% of control and 70% of worst cases were better tracked using FCM‐based fuzzy model than subtractive‐based fuzzy model. This was confirmed when subtractive clustering was used in the fuzzy section of ANFIS, as it operates as an overfitted version of SUB‐FIS due to the neural network capabilities in model learning. It should be considered that ANFIS takes a longer time for model configuration and update in comparison with the two other fuzzy modelers, owing to data processing in its neural network section.

Finally, an investigation was done concerning the performance of model selectivity algorithm. As derived from the validity results, the performance of this algorithm was not successful for all patients. This may be due to an insufficient number of cases in the training dataset, which is extremely critical to handle in terms of learning performance. But the decisions for best modeler selection in worst patient group were 80% correct, and final results when the selectivity option is active leads to the best performance overall, both in terms of median error and variability.

## V. CONCLUSIONS

Three different fuzzy logic‐based correlation models to predict tumor motion were investigated. The final analyzed results show adaptive selection of adequate fuzzy correlation models on a patient specific basis is feasible, despite the limited availability of model training data.

## ACKNOWLEDGMENTS

The authors acknowledge Sonja Dieterich for providing access to the clinical database. The research leading to these results has received funding from the European Community's Seventh Framework Programme ([FP7/2007‐2013] under grant agreement n° 215840‐2).
